# The Immature Heart: The Roles of Bone Marrow Stromal Stem Cells in Growth and Myocardial Repair

**DOI:** 10.2174/1874192400701010027

**Published:** 2007-11-23

**Authors:** Jun Luo, Minh Duong, Calvin Wan, Carolyn J Teng, Ray C.J Chiu, Dominique Shum-Tim

**Affiliations:** Division of Cardiothoracic Surgery, the Montreal General Hospital, MUHC, Canada

## Abstract

Studies have shown that adult bone marrow derived stem cells (MSCs) can participate in repair of myocardial injury in adult hearts, as well as in cardiac growth during fetal development in utero. Yet, no studies have evaluated the role of MSCs with respect to normal growth or tissue repair in immature hearts after birth. The present study examines whether MSCs may participate in the myocardial growth and injury in the post-natal immature hearts. MSCs were isolated from adult Lewis rats and labeled with Lac-Z gene using retroviral vectors. These MSCs were injected systemically into groups of neonatal (NB=2days-old), immature (B=30days-old) and adult (A=>3months-old) isogeneic Lewis rats. Additionally, left coronary artery ligation was carried out in subgroups of immature (BL) and adult (AL) rats one week after MSCs injection. The hearts were harvested serially from 2-days to 6-weeks, stained with X-Gal for labeled MSCs. Cardiomyocyte phenotypic expression was evaluated by immunohistological staining for Troponin I-C and Connexin-43. Labeled MSCs were found to home into the bone marrow in all rats of different developmental stages. They could be recruited from bone marrow into the infarcted site of myocardium only in groups AL and BL. They were also capable of differentiating into cardiomyocyte phenotype after myocardial injury. In contrast to that reported in the developing fetus, MSCs did not appear to contribute to the growth of non-injured hearts after birth. However, they can be recruited from the bone marrow and regenerate damaged myocardium both in the adult and in the immature hearts.

## INTRODUCTION

There is considerable evidence indicating that bone marrow stromal cells (MSCs) contain multipotent stem cells that can be induced to differentiate into cells of various phenotypes including cardiomyocytes, both **In vitro** [1] and *in vivo* [2]. Following acute myocardial infarction, experimental and early clinical studies reveal that these cells can survive and engraft into the peri-infracted myocardium where they can differentiate to participate in the repair process [3]. These studies had been done so far exclusively in mature animals and adult humans with no data on pediatric age group.

On the other hand, Liechty KW *et al. *[4] have shown that human MSCs engrafted and demonstrated site-specific differentiation after in utero transplantation in a sheep model. The donor MSCs could be detected for up to 5 months in various fetal tissues, suggesting that the implanted MSCs could participate in growth and organogenesis during fetal development. However, whether these pluripotent cells could participate in the continued growth of myocardium after birth remains undetermined.

In addition, the extensive experimental work and early clinical trials on MSC cell therapy for the treatment of cardiac patients with congestive heart failure (CHF) have not included any pediatric age group despite the fact that CHF represents a similarly pertinent problem in infants with congenital heart malformation or cardiomyopathy. In this study, we attempt to address the question of whether MSCs can contribute still in the growth of post-natal immature hearts as well as in the repair of injured myocardium in the pediatric age group.

## MATERIALS AND METHODS

### 1 Animal Model

Syngeneic Lewis rats with different body weight (180~200g for adult rats, Immature: 50~70g for immature rats, and 6~8g for newborn rats) were obtained from Charles River Laboratory (Laprairie Co, Quebec, Canada). These inbred rats were used as donors and recipients to simulate the autologous implantation of MSCs. All procedures were approved by the Animal Care Committee of McGill University and conformed to the “Guide for the Care and Use of Laboratory Animals” prepared by the Institute of Laboratory Animal Resources, National Academy Press (revised 1996), and the “Guide to the Care and Use of Experimental Animals” of the Canadian Council on Animals Care, published by the National Academy Press.

### 2 Isolation and Culture of MSCs

Isolation and primary culture of MSCs from the long bone of donor rats were performed as previously described using the Caplan’s method [5]. Briefly, the femoral and tibial bones were harvested from adult Lewis rats and both ends of the long bones were cut away from the diaphyses. The bone marrow was flushed out from the bone with complete medium, consisting of Dulbecco’s Modiﬁed Eagle’s Medium (DMEM) containing 10% fetal bovine serum and antibiotics: 100 U/ml penicillin G, 100 mg/mg streptomycin, and 0.25 mg amphotericin B (Gibco labora¬tories). The marrow plugs were then dissociated and the dispersed cells were centrifuged and resuspended twice in complete medium. These cells were then seeded into tissue culture dishes and cultured in an incubator (Forma Scientific, Inc.) with 5% CO_2_. Culture medium was replaced every three days and the non-adherent cells discarded. Following two passages, the attached cells were divided into three new flasks and cultured until the cell density of the colonies grew to approximately 90% conﬂuence. They were then transfected with reporter gene as described below.

### 3 Labeling of MSCs

After two passages, MSCs were transfected *ex vivo*, by a replication defective retrovirus containing the reporter Lac-Z gene that encodes for the bacterial-galactosidase enzyme produced by Lac-Z-GP AM12 amphotropic retrovirus producer cells [6]. MSCs were cultured with the supernatant from the Lac-Z-GP AM12 cells culture flasks with hexadimethrine bromide 4ug/ml. Twenty four hours after the last transfection, the transfection medium was removed and MSCs were maintained in complete medium containing 10% fetal bovine serum. Transfection efficiency was monitored in a subset of plates with X-gal staining to determine the percentage of cells expressing ß-gal activity. Briefly, the cells were rinsed with phosphate buffered saline (PBS) and ﬁxed with 1% glutaraldehyde at room temperature for 5 minutes. After replacing PBS with the staining solution (containing 1 mg/ml 5-bromo-4-chloro-3-indoyl-b-D-galactoside(X-gal), 2% dimethylsulfoxide, 20 mM K_3_Fe(CN)_6_, 20 mM K_4_Fe (CN)_6_.3H_2_O and 2 mM magnesium chloride), cells were incubated at 37°C, pH 7.8 to 8 and protected from light for 16h to detect the cells with ß-gal activities. The presence of blue-labeled cells was then conﬁrmed under inverted microscopy (BX-FLA, Olympus, Tokyo, Japan).

### 4 Preparation of Cells for Injection

Labeled MSCs were cultured with complete medium in 75 cm^2^ tissue culture flasks. On the day of transplantation, the cells in each flask were washed with 6 ml of Hank’s Basic Salt Solution (HBSS), and then trypsinized with 1 ml of trypsin-EDTA solution. The detached cell suspension was gathered in 2 ml of complete medium and the cell density was measured by a hematocytometer. Hence on, cells were collected by centrifuged at 1500 rpm for 5 min, and resuspended in complete medium without serum at a concentration of 4x10^7^cells/ml. The cell suspensions prepared in the tuberculin syringes were placed on ice until implantation.

### 5 Implantation of labeled MSCs

#### 5.1 Intravenous Injection for Implantation of MSCs in Adult and Immature Rats

Intravenous injection of labeled isogeneic MSCs was carried out through the tail vein in the adult (Group A, AL) and immature (Group B, BL) recipient rats. The recipient rats were placed in a glass canister where 3% isoﬂurane (MTC Pharmaceuticals, Cambridge, Ontario, Canada) at 1.5 l/min was introduced until anesthesia was achieved. General anesthesia was maintained by mask inhalation with 1.0% isoflurane at the same flow rate. Labeled cell suspension of 10^5^cells/g was injected into the tail vein of adult and immature rats with a 25-gauge or 28.5-gauge needle respectively.

#### 5.2 Intraperitoneal Implantation of MSCs in Newborn Rats

The 2 days-old newborn recipient rats received injection of labeled isogeneic MSCs through intraperitoneal route [4] because of the lack of venous access in this age group. A dose of 4x10^5^ cells/g labeled cells were injected into the left lower quadrant of the abdomen using a 28.5-gauge needle.

### 6 Experimental Groups

#### 6.1 Marrow Stromal Cell Transplant (n=90)

Labeled MSCs were injected systemically into groups of neonatal (NB= 2 days-old, n=20), immature (B= 30 days-old, n=20) and adult (A= >3 months-old, n=40) isogeneic Lewis rats.

The homing of labeled MSCs within the recipients’ bone marrow following systemic injection described above was examined at various time points ranging from 2 days to 6 weeks following the procedure. The bone marrow of the recipient rat was harvested as described earlier, plated and cultured in a similar fashion. When the cells were approximately 50–60% conﬂuent, they were stained for ß-gal activity as described above.

#### 6.2 Coronary Artery Ligation

One week after intravenous MSCs injection, 50% of the rats from adult (A) or immature group (B) were randomized into 2 subgroups (AL group, n=20, and BL group, n=10) and underwent coronary artery ligation. Briefly, anesthesia was introduced in a glass canister with 3% isoﬂurane at 1.5 l/min, and the rat was removed and placed in a restraining device after intubated with an intravenous catheter (16-gauge for adult rat or 18-gauge for immature rat) and ventilated at a tidal volume of 1.5~2.5 ml and a respiratory rate of 85 breaths/min. The heart was exposed via a left thoracotomy incision. The left coronary artery was ligated proximally with a 7-0 polypropylene suture. Satisfactory ligation with subsequent myocardial infarction was confirmed once an ischemic zone and hypokinetic area developed within the left ventricle of the heart. The incision was then closed in a sterile fashion and the rats survived until further studied as described below.

### 7 Histology and Immunohistochemical Studies

All rats were electively sacrificed at various time points from 2 days to 6 weeks following cell implant, as shown in Table1. The hearts were harvested and rinsed with PBS and perfusion ﬁxed in 2% paraformaldehyde in PBS. The staining for ß-galactosidase activity was performed as described above, but with the addition of 0.02% Nonidet P-40 and 0.01% deoxycholate to the staining solution. The gross heart specimens were stained over night at 37˚C and pH 7.8. After X-gal staining, they were embedded in paraffin and coronal sections of 5 µm thick were mounted on a set of gelatin coated glass slides such that serial sections could be used for different stains. A series of sections from each heart specimen were stained with hematoxylin and eosin and another serial sections from each heart were used for cardiomyocyte-specific immunohistochemical stainings, namely Connexin-43 (Zymed Laboratories Inc, San Francisco, CA), and Troponin I-C (Santa-Cruz Biotechnology Inc, Santa-Cruz, CA). Brieﬂy, after de-parafﬁnization, sections were placed in boiled citrate buffer (pH 6.0). After blocking in normal serum, sections were treated with the respective monoclonal antibodies overnight and with secondary antibodies the following day. Diaminobenzidine (DAB), which produces brown color, was then used as a chromogen for light microscopy. Counter-staining of sections by hematoxylin was also performed. Cells derived from implanted labeled MSCs were identiﬁed by their blue nuclei.

### 9 Statistical Analysis

All values are expressed as mean ± standard error of the mean (SEM). The unpaired Student’s t test was used to calculate the statistical significance of differences between the means of groups. A p-value of less than 0.05 was considered to be statistically significant.

### RESULTS

#### 1 X-Gal Staining of Normal Growing Hearts

**Fig. (1):**
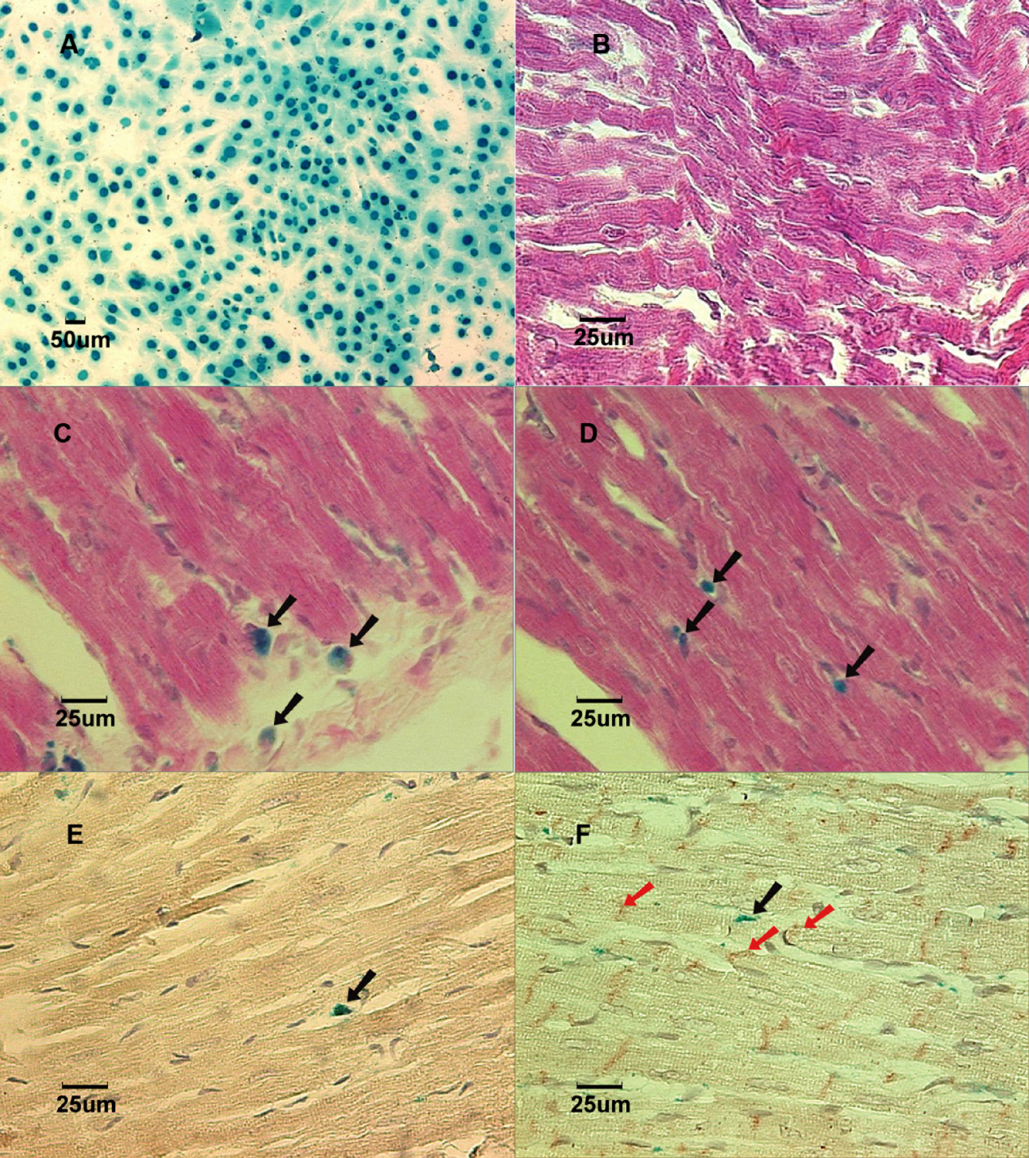
**A:**Lac-Z labeled MSCs (blue) prior to implantation in culture dish. **B:** (Group NB) Cross-section of myocardium 3 weeks after implantation showed no labeled MSCs in a normal growing heart. Stained with X-gal and counterstained with hematoxylin and eosin. **C:** (Group AL) Cross-section of myocardium 1 week after implantation showed Lac-Z positive cells in the infarcted area. Stained with X-gal and counterstained with hematoxylin and eosin. Black arrows show X-gal positively stained cells. **D:** (Group BL) Cross-section of myocardium 1 week after implantation showed Lac-Z positive cells in the infarcted area. Stained with X-gal and counterstained with hematoxylin and eosin. Black arrows show X-gall positively stained cells.** E:** (Group AL) Cross-section of myocardial scar 6 weeks after coronary ligation showed Troponin I-c positive cells. Brown color in the cytoplasm indicates positive stain. Counterstained with hematoxylin. Black arrow showed Lac-Z positive cell. **F:** (Group BL) Cross-section of myocardial scar 6 weeks after coronary ligation showed Connexin-43 positive cells in the peri-infarcted area. Counterstained with hematoxylin. The red arrows show Connexin-43 positive staining. Black arrow shows Lac-Z positive stained cells.

The MSCs usually become confluent in the culture flask after three to four passages of medium changes. At the end of Lac-Z gene transfection, the attached MSCs in the flask were nearly 100% labeled. The labeled cells stained with X-Gal solution shows blue nuclei under the inverted microscopy (Fig. **1A**).

The implantation of cells in Groups A, B, and NB had no mortality; and all the newborn rats tolerated the intraperitoneal injections. Two days after implantations, the homing of implanted MSCs into the bone marrow of the recipient rats were confirmed in all the groups at various time points: 2 days, 1 week, 3 weeks and 6 weeks.

**Fig. (2):**
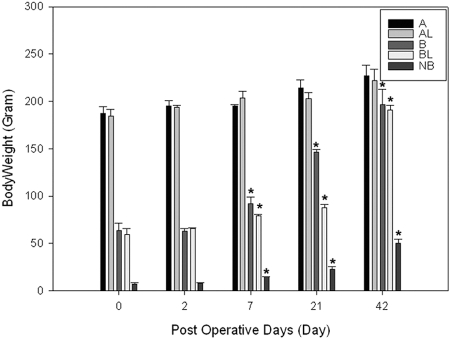
Body weight changes in groups A, B and NB. Note body weights in groups B, NB have significantly increased over 3 weeks period. * indicates p<0.05 compared with corresponding weight at day 0.

The body weight in group B and group NB had significantly grown from 63.1±6.7g to 207.8±7.6g and from 7.4±1.1g to 50.2±4.0g (p<0.05), respectively (Fig. **2**), at 6 weeks after MSC implantation (Table **2**). Yet, we were not able to detect any labeled cells in the heart samples taken from groups A, B, and NB (Fig.**1B**) at any time points ranging from 2 days to 6 weeks after implantations when stained with X-gal solution.

#### 2 X-Gal Staining of Hearts after Coronary Ligations

In the coronary artery ligation groups (AL and BL), all rats survived the surgical procedures throughout the duration of the experiments. All the heart samples from one week to six weeks after LCA ligations revealed a scar in the left anterior area of the left ventricle that corresponded to the left coronary artery distribution. After the heart samples were stained with X-gal solution, a consistent blue color could be seen in the infarcted myocardium in all LCA-ligated hearts, indicating that the labeled MSCs were recruited into the infarcted territory of the heart.

#### 3 Immunohistochemical Staining of Recipient Hearts after Injury

In the cross-sections of the injured heart samples taken from groups AL and BL, labeled MSCs could be clearly identified in all 30 hearts in the region of the scar. The labeled cells detected in these infarcted heart samples were characterized by undifferentiated appearance with round shape and large nucleus to cytoplasm ratio up until 3 weeks after implantation (Fig. **1C** and Fig. **1D** show 1 week samples). In the 6 weeks specimens, cell shape appeared elongated, resembling the adjacent cardiomyocytes. Immunohistochemical staining showed evidence of cardiomyogenic differentiation in some labeled cells with the expression of cardiomyocyte-specific Troponin I-C positive staining. The main constituent protein for the cardiac gap junctions, Connexin-43, was also detected in the x-gal labeled cells at 6 weeks specimens only in the coronary ligation groups (Fig. **1E** and Fig. **1F**).

## DISCUSSION

During cardiac development, the myocyte lineage is derived from the cardiogenic plates, which differentiate into contractile cells that rapidly divide and proliferate until the perinatal stage [7]. Liechty KW *et al. *provided evidence that there are other cell lineages in the bone marrow which can participate in cardiac organogenesis in sheep fetus [4]. Many reports have documented the presence of ‘non-hemato-poietic’ stem cell population in the bone marrow that can give rise to stromal supporting elements in both clinical [8] and experimental studies [2]. In contrast to that reported in the developing fetus [4], the current study was designed to evaluate the role of MSCs systemically delivered in the adult, immature and newborn rats. Our study suggests that MSCs administrated systemically have no significant toxicity to the normal growing individuals, are capable of homing to the bone marrow following intravenous [9] or intraperitoneal infusion. However, we did not find any evidence that labeled MSCs contributed to the growth of non-injured hearts at different developmental stages from neonates through adult ages. This may be related to the different mechanism of cardiogenesis during fetal and post-natal development. In contrast to the increase in myocardial mass prenatally by hyperplasia, the myocardial mass increases postnatally with normal growth are largely due to hypertrophy of the myocytes [3]. This implies that cell therapy will not be effective for promoting cardiac growth in patients with congenital heart defects after birth. Whether cell therapy will be able to prevent abnormal cardiac growth in utero warrants further investigation.

Since implanting bone marrow stem cells into the injured ventricle has been shown to improve cardiac function [10-12], numerous laboratories have focused on studying cellular cardiomyoplasty as a means of improving myocardial function due to heart failure [13-15]. Furthermore, randomized controlled clinical trial has been documented that intracoronary injection of autologous MSCs improved left ventricular systolic function in patients after acute myocardial infarction [9]. Although the mechanism by which stem cells improve cardiac function remains unclear, many studies have shown evidence that adult bone marrow stromal cells can participate in cardiac myogenesis [16], angiogenesis [17], and prevent cardiomyocyte apoptosis [12]. In our LCA ligation experiments, the results in the immature hearts are consistent with our previous findings in adult rodents [2], i.e. the marrow stromal cells residing within the bone marrow are able to be recruited, translocated through the blood stream, and migrated into the peri-infarct and infarcted segments of the myocardium.

One of the most intriguing properties of MSC is their ability to home to sites of inflammation or tissue damage. Although the steps responsible for this migration have yet to be fully elucidated, data in the literature suggests that it entails a 2-step process whereby stem cells first bind to their adhesive complexes around the injury zone, followed by local chemotaxis to the site of engraftment [18]. This phenomenon has been demonstrated in various settings including infarcted hearts [19], cerebral ischemia [20], and bone fractures [21]. In fact, Saito *et al. *from our laboratory were the first to demonstrate that MSCs administered intravenously engraft within the infarcted myocardium, whereas those injected in healthy rats, home to the bone marrow [19]. In another study, Kraitchman *et al.* [22] showed the remarkable specificity with which MSC can home to infarcted regions.

SDF-1 and its receptor CXCR4 are required for stem cells to home to the bone marrow. Their role in coronary artery disease is less clear. Previous studies have shown the expression of SDF-1 in atherosclerotic plaques, its upregulation in the heart early after MI as well as the increase in neovascularization following its exogenous expression [18]. Askari *et al. *further reinforced the role of SDF-1 in stem cell homing in a study whereby cardiac fibroblasts expressing SDF-1 were transplanted into the infarcted regions of rat hearts [23]. After using granulocyte stimulating factor (G-CSF) to mobilize stem cells, a significant homing of c-kit cells to the injured area as well as an improved cardiac function was found in treated animals. Orlic and his group have also demonstrated the upregulation of MSC homing and differentiation with the use of G-CSF [24]. In this study, a 250-fold increase in the levels of Lin-/c-kit+ cells as well as an improvement in the ventricular function were found in rats that were pretreated with G-CSF and stem cell factor (SCF). A similar finding was obtained when granulocyte-macrophage stimulating factor (GM-CSF) was used [25]. Although the exact mechanism is yet to be understood, Harada *et al. *recently showed that this G-CSF-mediated stem cell mobilization and improvement in cardiac function occur through the activation of the Jak/Stat pathway in the cardiomyocytes, hence inducing a number of anti-apoptotic proteins and angiogenic factors [26].

Other than SDF-1, SCF is also involved in the regulation of stem cells migration by binding to its tyrosine kinase receptor, c-kit, which is expressed on a variety of stem cell lines [27]. This is confirmed by further studies showing the role of SCF in the induction of the expression of CXCR4 on human CD34+ cells resulting in an increase in their migration in response to SDF-1116. A wide variety of chemokines have actually been shown to modulate such migration. However, the largest response was seen with α and β SDF-1141. Furthermore, it is important to realize that although SDF-1 is required in stem cell mobilization to the injured site, it is not singularly sufficient, hence reflecting the need for additional factors. In fact, patients with acute ischemic injury have elevated levels of many factors other than SDF-1 including MMP-2, MMP-9, ICAM, and VCAM [28].

Cell-to-cell interactions as well as other environmental factors involving a combination of paracrine growth factors promote stem cell migration and differentiation. Eghbali-Webb has recently reviewed the role of cardiac fibroblasts in regulating myocardial regeneration by the release of various soluble factors within the extracellular matrix such as VEGF, FGF, TGF-β1, PDGF, and MMPs, highlighting the coordinated cell-to-cell and cell-to-environment interactions [29]. It is also possible that the hypoxia following an ischemic insult can enhance the expression of some adhesion molecules and thus facilitates MSC migration. For instance, the increase in MMP-9 level following the use of mobilizing agents such as SDF-1, VEGF and G-CSF or after a myocardial infarction, leads to an up-regulation of soluble kit, which ultimately results in an increase in MSC mobilization and proliferation [30, 31].

It is conceivable that the signaling pathways for normal growth and tissue injury are very different that may explain the different responses of the MSCs following myocardial injury and developmental growth. After migrating to the injured site, these labeled marrow stromal cells appeared to differentiate into various phenotypes, including cardiomyocytes. Since surgical interventions may cause damage to the myocardium [24], cellular cardiomyoplasty may be useful for repairing the heart muscle after cardiac surgery. Our data are consistent with the notion that bone marrow serves as a reservoir for potential cardiac precursor cells, which can generate cardiomyocytes de novo in the presence of proper signal and stimulation.

This study has several limitations. First, we have not examined the issue of “cell fusion”. Although transdifferentiation of MSCs into cardiomyocytes had been reported *in vivo* [4,10,16] and **In vitro** [32], Murry, C. E.*et al. *[33] and Nygren, J. M. *et al. *[34] showed that haematopoietic stem cells could adapt the cardiomyocyte phenotype by spontaneous fusion with the host cells. Recently, Kajstura, J. *et al. *[35] and others have reported evidence that bone marrow cells could indeed differentiate into cardiac cell lineages after infarction, independently of cell fusion. Matsuura *et al. *. [36] reported that even after cardiomyocyte fused with surrounding cells, they could reenter G2-M phase in the cell cycle, and maintained their cardiomyocyte phenotype. Wager and Weissman [37] proposed that cell fusion-mediated regeneration might be considered one of the physiological mechanisms of repair. These studies suggested that augmented cell fusion in the diseased heart may contribute to the maintenance and replenishment of cardiomyocytes, as in the cardiomyocytic transdifferentiation of implanted cells.

Second, the adherent subpopulations of bone marrow cells under culture, known as marrow stromal cells (MSCs), contain different cell surface markers. In this study, we did not fractionate and purify the cells by such specific cell markers [38]. However, unlike that of differentiated cells, the cell markers expressed by multipotent cells undergoing differentiation may change with time and micro-environment. Therefore, considerable controversy persists as to the usefulness of such markers to identify the differentiating cell population contributing to myocardial cell therapy. Other limitations of this study include: 1) the lack of quantitative study to document the proportion of implanted cells which survived and differentiated, and 2) no attempts to investigate the functional effects of such therapy in immature hearts. Yet, numerous reports had documented the improvement in ventricular function associated with anatomical and immunohistochemical findings similar to those observed in our study in adult hearts [15, 39, 40].

In conclusion, although further studies are needed, our findings have shed some lights both on the potential and limitations in the use of marrow stromal cell therapy for the repair of injured hearts in both adults and immature age groups [41], as well as their different response for normal growth.

## Figures and Tables

**Table 1:** The Intervals Between MSCs Injection and Harvesting of Cardiac Specimen

**Group**	**2 days**	**1 week**	**3 weeks**	**6 weeks**	**n**
A	4	4	4	8	20
AL	4	4	4	8	20
B	2	2	2	4	10
BL	2	2	2	4	10
NB	4	4	4	8	20

A=Adult; AL=Adult and LCA Ligated; B=Immature; BL=Immature and LCA Ligated; NB=New Born.

**Table 2:** 

**Group**	**N**	**Age**	**Bodyweight (g)**	**Number ofMSCs implanted (X10^6^)**	**Implantation Route**	Heart^*^
A	20	>3M	189.7±8.8	20	IV	-
AL	20	>3M	185.3±7.8	20	IV	+
B	10	30D	63.1±6.7	6	IV	-
BL	10	30D	61.0±6.7	6	IV	+
NB	20	2D	7.4±1.1	3	IP	-

A=Adult; AL=Adult and LCA Ligated; B=Immature; BL=Immature and LCA Ligated; NB=New Born; M=Month; D=Days; *: Presence (+) or Absence (-) of labeled MSCs in the myocardium.
